# Supplementary material for: Identification of Novel Inhibitors of Starch Excess 4 (SEX4)
Source: Life (Basel). 2024 Dec 20;14(12):1686. doi: 10.3390/life14121686 (PMC11678766; doi:10.3390/life14121686)
Supplement: Supplementary file 1 [file life-14-01686-s001.zip › life-3326348-supplementary.pdf]

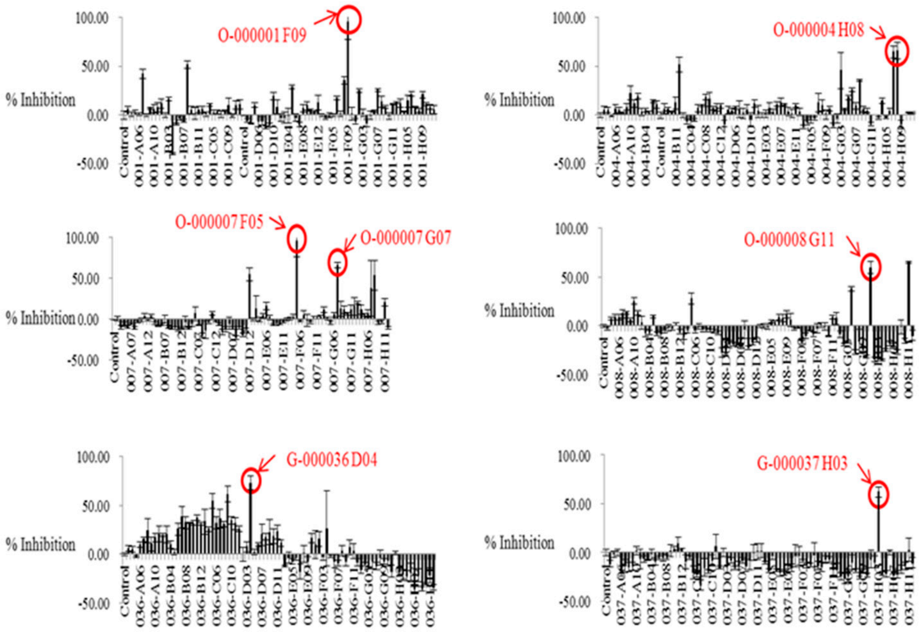

Figure S1. Identification of seven compounds among ~1840 compounds through SEX4's inhibitor screening using *p*NPP assay. % inhibition means the relative decreasing percentage of each assay with 0.2 mM compounds compared to the activity value of control assay without compounds. The red circle means selected compounds showing an inhibition rate of more than 50% and confirmed by reproduction experiment. The plate numbers are shown in red next to the red circle with an arrow.

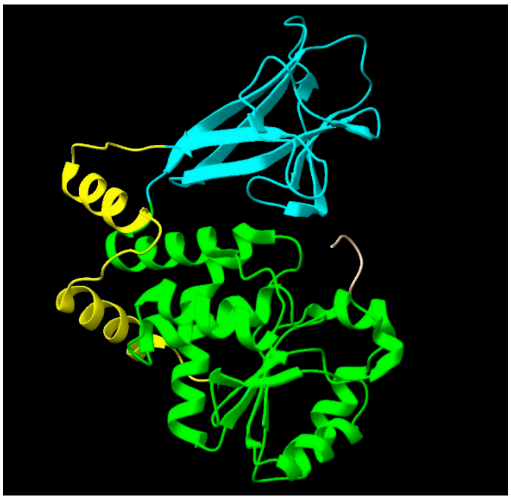

(a)

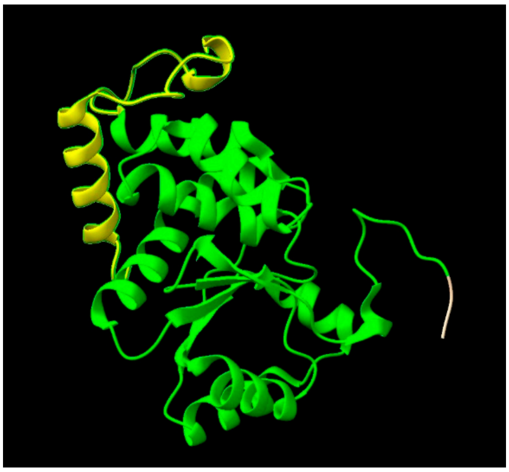

(b)

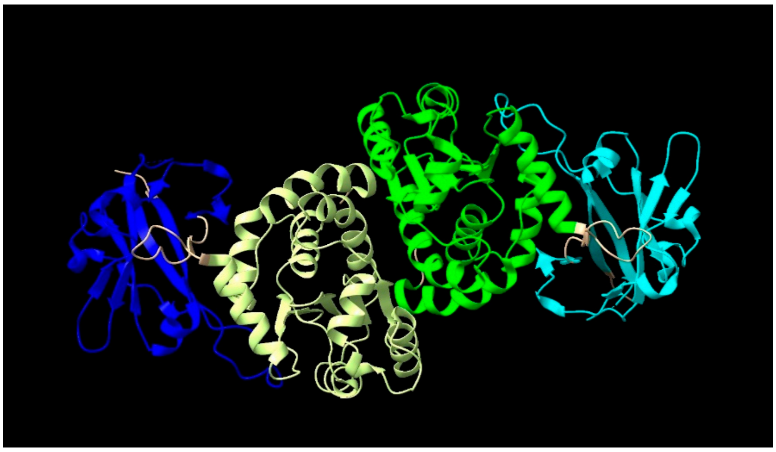

(c)

Figure S2. The representative structural images of SEX4 (a, PDB ID: 4PYH), LSF2 (b, PDB ID: 4KYQ), and Laforin (c, PDB ID: 4RKK). In a, the green color means Dual Specificity Phosphaase (DSP) domain (residues 90 to 252), the sky blue color is Carbohydrate Binding Module (CBM) domain (residues 253 to 338) and yellow color is C-terminal (CT) motif (residues 339 to 379) of SEX4. In b, the green color means DSP domain (residues 79 to 244) and yellow color is CT motif (residues 245 to 282) of LSF2. In c, the blue color means CBM domain of chain A (residues 1 to 124), the bright yellow color is DSP domain

of chain A (residues 138 to 322), the green color is DSP doamin of chain C (residues 138 to 322) and the sky blue is CBM domain of chain C (residues 1 to 124) of dimeric Laforin.
